# The Effect of a Hydrolyzed Polysaccharide Dietary Supplement on Biomarkers in Adults with Nonalcoholic Fatty Liver Disease

**DOI:** 10.1155/2018/1751583

**Published:** 2018-05-03

**Authors:** John E. Lewis, Steven E. Atlas, Oscar L. Higuera, Andrea Fiallo, Ammar Rasul, Ashar Farooqi, Olga Kromo, Laura A. Lantigua, Eduard Tiozzo, Judi M. Woolger, Sharon Goldberg, Armando Mendez, Allan E. Rodriguez, Janet Konefal

**Affiliations:** ^1^Department of Psychiatry and Behavioral Sciences, University of Miami Miller School of Medicine, Miami, FL, USA; ^2^Rheumatic Wellness Institute, LLC, South Miami, FL, USA; ^3^Department of Medicine, University of Miami Miller School of Medicine, Miami, FL, USA; ^4^Glow Health PA, Bay Harbor Islands, FL, USA; ^5^Department of Family Medicine and Community Health, University of Miami Miller School of Medicine, Miami, FL, USA

## Abstract

The primary objective of the study was to evaluate the effect of a hydrolyzed polysaccharide, Rice Bran Arabinoxylan Compound (RBAC), on biomarkers in adults with nonalcoholic fatty liver disease (NAFLD). A 90-day randomized double-blind placebo-controlled trial examined the effect of RBAC on complete blood count, liver enzymes, lipids, oxidative stress markers, cytokines, and growth factors. Twenty-three adults with NAFLD were enrolled and randomly assigned to one of the two study conditions (*n* = 12 RBAC and *n* = 11 placebo) and consumed 1 gram/day of either compound for 90 days. Subjects were assessed at baseline and 45 and 90 days. No adverse effects were reported. Alkaline phosphatase significantly decreased (−3.1%; SD = 19.9; *F*[1,19] = 5.1, *p* = 0.03) in the RBAC group compared to placebo. Percent monocytes (17.9%; SD = 18.3; *F*[1,19] = 5.9, *p* = 0.02) and percent eosinophils (30.6%; SD = 30.5; *F*[1,19] = 12.3, *p* < 0.01) increased in the RBAC group. IFN-*γ* (156%; SD = 131.8; *F*[1,19] = 4.2, *p* = 0.06) and IL-18 (29.1%; SD = 64; *F*[1,19] = 5.3, *p* = 0.03) increased in the RBAC group compared to placebo. Other improvements were noted for platelets, neutrophils, neutrophil-lymphocyte ratio, *γ*-glutamyl transferase, and 4-hydroxynonenal. RBAC had beneficial effects on several biomarkers that add to the known immunomodulatory activities of RBAC, which may be promising for people with NAFLD.

## 1. Introduction

 Nonalcoholic fatty liver disease (NAFLD) describes a spectrum of diseases characterized by hepatic fat accumulation [1]. NAFLD ranges from simple steatosis to nonalcoholic steatohepatitis (NASH) categorized with inflammation, fibrosis, and cirrhosis [2]. Approximately, 20–30% of the population has NAFLD [3, 4], while the prevalence of NASH is roughly 2-3% [4, 5]. NAFLD has risen in prevalence in proportion to obesity, type 2 diabetes, and dyslipidemia [2], and NAFLD is an emerging epidemic. The goal of NAFLD management is to improve steatosis and prevent fibrosis. Currently, this is accomplished via lifestyle interventions, medical treatments, alternative therapies, and surgery aimed at modifying key NAFLD risk factors: obesity and insulin resistance [1, 6–8].

Nutritional optimization through dietary supplementation may offer an alternative treatment strategy for NAFLD. Many studies have shown that a potent hydrolyzed polysaccharide, Rice Bran Arabinoxylan Compound (RBAC), possesses a biologic response modifier effect on immune system function, particularly natural killer (NK) cell activity [9–15]. For example, two separate rat studies have demonstrated the effectiveness of RBAC on various biomarkers after d-galactosamine- (Ga1N-) induced acute liver disease, which is essentially a model of hepatitis in humans [9, 10]. This form of hepatitis was shown to be suppressed by RBAC. The protective mechanism was mediated in part by downregulation of interleukin-18 (IL-18) in the first study [9]. The second study showed that nuclear factor-*κ*B (NF-*κ*B) and CD14+ were involved in the suppressive action of RBAC on GalN-induced hepatitis [10]. The serum activity of transaminases (alanine transaminase [ALT] and aspartate transaminase [AST]) was significantly higher after GalN treatment, but the changes were attenuated by RBAC. Furthermore, GalN-induced inhibitor of *κ*B kinase degradation appeared to be prevented by RBAC, and associated suppression of NF-*κ*B activation was noted. Additionally, RBAC significantly inhibited CD14+ mRNA expression [10]. RBAC is also likely to possess antioxidant capabilities, as it has been shown to enhance macrophage phagocytic activity and nitric oxide release and scavenge free radicals in a dose-dependent manner [11, 12]. RBAC has been shown to have significant immunomodulatory and net anti-inflammatory activity in several studies, particularly in cancer [13, 14]. In our lab, we previously showed that RBAC enhanced NK cell cytotoxicity, demonstrated changes in 9 out of 12 cytokines and growth factors, and was well-tolerated and safe among a sample of healthy adults [15].

Based on the prior studies, dietary supplementation in NAFLD may confer favorable metabolic and immunological effects, yet we are aware of no study that has investigated the effect of a polysaccharide nutritional supplement like RBAC on related biomarkers in patients with this disease. The purpose of this study is to determine the effect of 90 days of RBAC treatment on biomarkers in patients with NAFLD.

## 2. Materials and Methods

### 2.1. Subjects

The study was conducted with the approval of the University of Miami Institutional Review Board for human subject research (registry: https://www.clinicaltrials.gov/ct2/show/NCT02568787). Potential subjects were initially identified from physician referrals, the Medical Wellness Center, and the Departments of Psychiatry and Behavioral Sciences and Medicine at the University of Miami Miller School of Medicine, where the data were collected. Recruitment began in June 2016 and ended in February 2017 after target enrollment was achieved. Inclusion criteria were as follows: (a) age 18 or older; (b) confirmed NAFLD diagnosis by referring or treating physician; (c) stable medication regimen during the intervention; (d) planning to maintain current medication during the course of the intervention; (e) previous dietary supplement usage of similar polysaccharide formulas permitted, but must be discontinued 2 weeks before and for the duration of the trial; (f) willing to follow recommendations for assessment and intervention study protocol; and (g) able to provide informed consent. Exclusion criteria were as follows: (a) currently enrolled in another research trial for similar investigative nutritional therapies; (b) known allergy to rice, rice bran, mushrooms, or related food products; (c) any gastrointestinal disorders that could lead to uncertain absorption of the study supplement; (d) use of lipid-lowering agent 3 months prior to study enrollment; (e) current immunomodulator use; (f) active chemotherapy; (g) severe anemia or other medical condition that would preclude a safe blood draw; (h) bleeding disorder; or (i) active pregnancy or attempting conception.

Thirty-nine subjects were screened for inclusion and exclusion criteria. Thirteen were ineligible to participate in the study, and three were eligible but did not enroll. Thus, twenty-three subjects met the criteria and were enrolled in the study after signing the informed consent and HIPAA privacy forms prior to study entry. The participants were assigned by study staff using a simple randomization procedure to one of two conditions: (a) RBAC (*n* = 12) or (b) placebo (*n* = 11), using a random permutations table created by the principal investigator (JEL). All subjects and investigators were blinded to the treatment condition and remained blinded until after data analysis. Placebo and supplements were provided by Daiwa Health Development (Gardena, CA, USA) labeled as Protocol A and Protocol B. Only a staff member at Daiwa Health Development knew the assignment of treatment to Protocol A or B. After randomization, participants were scheduled for assessments at baseline and 45- and 90-day follow-up (±7 days). Blood was drawn at each time point to assess the biological markers. Subjects were compensated $50 for completing the assessment at each time point. Three participants dropped out of the study at 45 days, and thus 20 subjects completed the study. Data collection was completed in May 2017.

### 2.2. Intervention

All subjects were instructed to take 2 capsules 1 time per day (1 g/day total) for the 90-day intervention period. Subjects were advised to not modify dietary or physical activity habits or prescription medication use. Subjects were also instructed not to consume any known immune-active pharmaceutical agents or any dietary supplements containing mushroom products for two weeks prior to having the baseline assessment and until the conclusion of the 90-day study period. Consuming RBAC is similar to rice bran and should be tolerated like other common foods. We are not aware of any documented side effects of RBAC, and our first study with this product reported no adverse events [15]. RBAC is a water-soluble extract of rice bran that has been hydrolyzed by an enzyme complex extracted from shiitake mushroom. In addition, RBAC contains microcrystalline cellulose, hypromellose, sucrose fatty acid ester, gellan gum, and potassium acetate. Each capsule contained 500 mg of RBAC. The placebo capsules were indistinguishable from the RBAC but contained cellulose.

### 2.3. Outcomes and Assessments

Each participant completed a basic demographics and medical history questionnaire at baseline. Subjects were also asked to list their current medications and note any changes in type or amount during the course of the study. Criteria used to select the assessment instruments included (a) appropriateness for the population; (b) ease of administration and scoring; (c) experience administering these measures; and (d) employment of measures involving a multimethod (i.e., self-report and biological values) approach to enhance the validity of the overall assessment.

### 2.4. Blood Draw Procedures

Participants abstained from caffeine and alcohol consumption for 24 hours before testing, which was conducted in the postabsorptive state following an overnight (12-hour) fast. A sample of blood (30 mL) was drawn at each visit.

### 2.5. Kidney Function, Liver Enzymes, and Oxidative Stress Markers

Bilirubin, creatinine, protein, albumin, liver enzymes (AST, ALT, and alkaline phosphatase [ALP]), AST/ALT ratio, 4-hydroxynonenal, malondialdehyde, and *γ*-glutamyl transferase (GGT) were assessed at each time point.

### 2.6. Lipids

Total, low-density lipoprotein (LDL), very low-density lipoprotein (VLDL), and high-density lipoprotein (HDL) cholesterol and triglycerides were assessed at each time point.

### 2.7. Complete Blood Count

The standard complete blood count (CBC) was measured at each time point. Neutrophil-to-lymphocyte ratio (NLR; absolute count) was calculated as a marker that is predictive of chronic inflammation in cardiovascular disease and cancer and may be useful in evaluating disease status in NAFLD as well [16–18].

### 2.8. Immunological Variables

Proinflammatory cytokines (tumor necrosis factor- [TNF-] *α*, TNF-*β*, interleukin- [IL-] 1*α*, IL-1*β*, IL-6, TNF RI, and IL-18), T helper- (Th-) 1 cytokines (interferon- [IFN-] *γ*, IL-12, IL-2, IL-15, and TNF RII) and IL-8 and Th-2, Th-17, and anti-inflammatory cytokines (IL-4, IL-5, IL-17, IL-23, IL-10, and IL-13) were measured at each assessment.

### 2.9. Biomarker Assays

Blood samples were collected into ethylene diamine tetra acetic acid (EDTA) anticoagulant tubes. Plasma was separated within 2 hours of collection and either analyzed on the same day (routine chemistries) or stored at −80°C until assayed (cytokines and oxidative stress markers). Routine chemistries, including bilirubin, creatinine, protein, albumin, AST, ALT, ALP, GGT, and all lipids, were measured on a Roche 6000 chemistry analyzer (Roche Diagnostics, Indianapolis, IN, USA) using manufacturers reagents and following all instructions for instrument set-up and assay procedures. All chemistry tests had inter- and intra-assay CVs < 4.5%. CBC was measured in whole blood on the day of collection using a Beckman Coulter UniCel DxH 600 hematology analyzer. Malondialdehyde and 4-hydroxynonenal were measured by ELISA using kits obtained from Cell Biolabs, Inc. (San Diego, CA, USA). Samples for malondialdehyde and 4-hydroxynonenal were frozen at −80°C until assayed, and all samples were analyzed on the same day.

The cytokines and growth factors were measured in plasma using Quansys reagents and ELISA kits (Quansys Biosciences, Logan, UT, USA) in the same way as reported previously [19]. The ranges of the cytokine and growth factor concentrations used in the standard calibration samples were adjusted for each analyte along with sample exposure time.

### 2.10. Descriptive and Control Variables

Demographics such as age, race/ethnicity, socioeconomic status, education, employment status, and current living situation were assessed at baseline. Medical history was measured with a questionnaire that included history of surgery, hospitalization, respiratory diseases, diabetes, coronary artery disease, neurodegenerative disorders, mental health, cancer, use of prescription and over-the-counter medications, and alcohol and tobacco consumption.

### 2.11. Adverse Events

Participants were monitored until the end of the study. Potential side effects were explained to each participant during informed consent.

### 2.12. Compliance

Compliance was measured using a modified version of the 8-item Morisky Medication Adherence Scale (MMAS-8). MMAS-8 is a generic, validated, self-reported measure of medication-taking behavior that does not target a specific age, disease, or treatment group.

### 2.13. Statistical Analyses

Frequency and descriptive statistics were calculated on all variables. Independent samples *t*-tests and chi-squares were utilized to evaluate differences in sociodemographic and clinical history characteristics between groups at baseline. Percent change was calculated for the difference between (a) baseline and 45-day follow-up, (b) baseline and 90-day follow-up, and (c) 45- and 90-day follow-up for all biomarkers. Then, the percent change dependent variables were evaluated in one-way analysis of variance to compare differences between the placebo and RBAC groups. IBM SPSS Statistics 24 for Windows (IBM, Inc., Chicago, IL, USA) was used for statistical analyses, and *α* < 0.05 was considered statistically significant.

## 3. Results

### 3.1. Sociodemographics, Comorbid Disorders, and Medication Use

See Table 1 for the descriptive information of the sample for age, gender, race/ethnicity, education, and marital status, which were all nonsignificantly different between the RBAC and placebo groups. The most prevalent comorbid conditions were hypertension (*n* = 11 [48%]), dyslipidemia (*n* = 11 [48%]), hypertriglyceridemia (*n* = 9 [39%]), and migraines (*n* = 6 [26%]), and the differences between groups for these disorders were insignificant. Subjects were taking an average of 4.1 prescription (SD = 4.3,* R* = 0, 17) and 1.3 over-the-counter (SD = 1.2,* R* = 0, 4) medications.

### 3.2. Compliance to the Protocol

According to the MMAS-8 total scores, 83% of the sample had medium to high compliance at 45 days, and 61% of the sample had medium to high compliance at 90 days. During the entire study period, no adverse event was reported.

### 3.3. Analysis of Liver Enzymes, Kidney Function, Lipids, and Oxidative Stress Markers

Table 2 shows the descriptive statistics for liver enzymes, AST/ALT ratio, GGT, albumin, non-HDL cholesterol, and 4-hydroxynonenal. The percent change in ALP from baseline to 90 days was statistically significant (*F*[1,19] = 5.1, *p* = 0.03), as the placebo group increased by 16.9% (SD = 19.7), while the RBAC group decreased by 3.1% (SD = 19.9). The percent change in ALP from baseline to 45 days was a statistical trend (*F*[1,19] = 3.6, *p* = 0.07), as the placebo group increased by 9% (SD = 11.7), whereas the RBAC group decreased by 3.7% (SD = 16.9). The percent change in albumin from baseline to 90 days was a statistical trend (*F*[1,19] = 3.1, *p* = 0.09), as the placebo group increased by 1.6% (SD = 3.0), whereas the RBAC group decreased by 1.0% (SD = 3.5). The percent change in AST/ALT ratio from 45 to 90 days was a statistical trend (*F*[1,19] = 4.1, *p* = 0.06), as the placebo group increased by 14.2% (SD = 22.6), whereas the RBAC group decreased by 2.1% (SD = 13.2). Although not statistically significant, GGT decreased in the RBAC group from 67 IU/L at baseline to 47 IU/L at 90 days, while it increased in the placebo group from over 78 IU/L at baseline to 98 IL/L at 90 days. The percent change in the non-HDL cholesterol from baseline to 45 days was a statistical trend (*F*[1,19] = 3.3, *p* = 0.08), as the placebo group decreased by 5.8% (SD = 7.6), whereas the RBAC group increased by 5.3% (SD = 16.8). Although not statistically significant, 4-hydroxynonenal decreased in the RBAC group from baseline (121 *μ*g/mL) to 90 days (76 *μ*g/mL), whereas the placebo group increased from 145 *μ*g/mL at baseline to 911 *μ*g/mL at 90 days.

### 3.4. Analysis for While Blood Cell Subsets and Platelets

Table 3 shows the descriptive statistics for white blood counts, neutrophils, lymphocytes, NLR, monocytes, eosinophils, and platelets. The percent change in white blood cells from baseline to 90 days was significantly different (*F*[1,19] = 6.4, *p* = 0.02), as the placebo group increased by 12.7% (SD = 9.8), whereas the RBAC group remained the same at 0.1% (SD = 12.0). The percent change in monocytes (%) from 45 to 90 days was significantly different (*F*[1,19] = 5.9, *p* = 0.02), as the placebo group decreased by 2.2% (SD = 18.7), whereas the RBAC group increased by 17.9% (SD = 18.3). The percent change in eosinophils (%) from baseline to 45 days was significantly different (*F*[1,19] = 5.0, *p* = 0.03), as the placebo group decreased by 11.8% (SD = 39.8), whereas the RBAC group increased by 26.2% (SD = 36). The percent change in eosinophils (%) from baseline to 90 days was significantly different (*F*[1,19] = 12.3, *p* < 0.01), as the placebo group decreased by 12.7% (SD = 39.8), whereas the RBAC group increased by 30.6% (SD = 30.5). The percent change in total monocytes from 45 to 90 days was a statistical trend (*F*[1,19] = 3.3, *p* = 0.08), as the placebo group decreased by 0.2% (SD = 21.8), whereas the RBAC group increased by 18.7% (SD = 24.3). Although not statistically significant, neutrophils (%) decreased in the RBAC group from baseline (53.1) to 90 days (48.5), while no change occurred in the placebo group from baseline (56.7) to 90 days (56.6). Additionally, the NLR decreased slightly in the RBAC group from baseline (1.5) to 90 days (1.4), whereas it remained constant at 1.8 from baseline to 90 days in the placebo group. The percent change in platelets (10^3^/*μ*L) from baseline to 90 days was significantly different (*F*[1,19] = 5.7, *p* = 0.02), as the placebo group increased by 13.1% (SD = 21), whereas the RBAC group decreased by 5.1% (SD = 12.8). The percent change in platelets (10^3^/*μ*L) from baseline to 45 days was a statistical trend (*F*[1,19] = 3.5, *p* = 0.07), as the placebo group increased by 6.1% (SD = 17.8), whereas the RBAC group decreased by 6.5% (SD = 12.4).

### 3.5. Analysis for Cytokines and Growth Factors

Table 4 shows the descriptive statistics for the cytokines and growth factors. The percent change in IL-18 from 45 to 90 days was significantly different (*F*[1,19] = 5.3, *p* = 0.03), as the placebo group decreased by 22.9% (SD = 22.7), whereas the RBAC group increased by 29.1% (SD = 64). Although not statistically significant, IL-18 declined from 446 pg/mL at baseline to 221 pg/mL at 90 days for the RBAC group, whereas the placebo group slightly increased from baseline (256 pg/mL) to 90 days (263 pg/mL). The percent change in IL-6 from baseline to 45 days was a statistical trend (*F*[1,19] = 3.4, *p* = 0.08), as the placebo group increased by 200.5% (SD = 156.5), whereas the RBAC group decreased by 68.9% (SD = 160.2). The percent change in IL-2 from baseline to 90 days was significantly different (*F*[1,19] = 6.7, *p* = 0.02), as the placebo group increased by 76.5% (SD = 108.1), whereas the RBAC group decreased by 13.3% (SD = 37.8). The percent change in IFN-*γ* from baseline to 90 days was a statistical trend (*F*[1,19] = 4.2, *p* = 0.06), as the placebo group increased by 36.5% (SD = 128.9), whereas the RBAC group increased by 156% (SD = 131.8). The percent change in TNF RII from 45 to 90 days was a statistical trend (*F*[1,19] = 3.6, *p* = 0.07), as the placebo group decreased by 22% (SD = 43.2), whereas the RBAC group increased by 7.1% (SD = 24.9).

## 4. Discussion

The incidence and prevalence of NAFLD are rising worldwide with a paucity of available treatment approaches, aside from reducing liver fat content via weight loss [20–23]. Thus, it is important to explore promising dietary supplement or nutritional interventions such as RBAC.

The hepatic enzymes, that is, AST, ALT, and ALP, constitute a classic group of tests to determine hepatocyte integrity, as elevated values suggest varying liver damage [24]. In the current study, ALP decreased in the RBAC group at 45 and 90 days, whereas in the placebo group it increased, which is consistent with a rat study showing that ALT and AST were reduced by RBAC after GalN treatment [10]. The present finding is important, given that an increasing ALP level portends potential liver damage [24] and is related to steatosis and fibrosis [25].

The increases in percentages of eosinophils and monocytes at 90 days in the RBAC group suggest an immunomodulatory (or a short-term immunostimulatory increase) response, which parallels the effects that have been previously noted on various white blood cell counts or percentages in response to treatment with this polysaccharide [26–28]. In this case, a short-term mild inflammatory response could be beneficial to protect hepatocytes from damage, enable tissue damage restoration, and support the overall effort to achieve homeostasis, as opposed to chronic inflammation that would enhance liver injury and promote steatosis and cirrhosis [29]. Simultaneously, we observed nonsignificant decreases in both neutrophils (%) and NLR, which are clinically important. Neutrophils are the primary cells of the innate immune system, and their predominance is likely related to oxidative stress, initiation of matrix metalloproteinases, and a heightened proinflammatory response, for example, activation of Th-17 molecules [16, 30–33]. In the current study in the RBAC group, as neutrophils decreased from baseline to 90 days, so too did IL-17, which is consistent with other studies showing that neutrophils are important for activating IL-17 in the liver, particularly for fibrosis [16, 34, 35]. A higher NLR has been linked to poorer outcomes in NAFLD and NASH and is also predictive of mortality [16, 36, 37]. Additionally, GGT and 4-hydroxynonenal also decreased in the RBAC group, whereas both increased in the placebo group, although nonsignificantly. GGT decreased to a value within the normal range (<50 IU/L), which is clinically significant and of particular interest to NAFLD patients, given GGT's linear relationship to increasing levels of hepatobiliary disease and different neoplasms [38–41]. Additionally, both ALP and GGT decreased in the RBAC group, which is clinically significant as their movement together is used as an indicator of biliary obstruction [42, 43], giving more credibility to the current findings. Increased 4-hydroxynonenal is indicative of greater oxidative stress and in addition to liver diseases its production is implicated in the pathogenesis of cancer and neurodegenerative disorders [44–47]. Platelets are an acute phase reactant [48, 49], so the decrease observed in the RBAC group could possibly be an indicator of a beneficial effect, even though the values were within the normal range. Thus, the improvements in neutrophils, NLR, GGT, 4-hydroxynonenal, and platelets all suggest valuable clinical implications for this patient population.

IL-18 and the percentage of monocytes significantly increased from 45 to 90 days in the RBAC group compared to placebo. IL-18 is generally produced by monocytes and macrophages as an inflammatory response to either pathogens or stress [50, 51]. Additionally, the increase in IFN-*γ* in the RBAC group may be at least partially explained by the change in IL-18, as it has been shown that IL-18 initiates Th-1 polarization and stimulates NK cells, which together create large amounts of IFN-*γ* [50, 52, 53].

While IL-18 significantly increased from 45 to 90 days, the overall level from baseline to 90 days decreased in the RBAC group, although not significantly. The decrease in IL-18 is consistent with a previous rat study on RBAC and d-Ga1N-induced liver disease, which showed that RBAC suppressed a model of hepatitis by at least partially downregulating IL-18 [9]. Thus, the results of the current study suggest a complex interplay between monocytes, IL-18, and IFN-*γ* in response to our subjects taking RBAC.

### 4.1. Limitations

The results of the current study were likely limited due to several factors. We did not enroll only patients who had liver biopsies due to the difficulty of finding an adequate number of potential subjects willing to undergo this procedure, so the referring physician's diagnosis of NAFLD was based on a number of clinical factors that contain some error, for example, body composition and liver enzyme tests. A homogenous sample of subjects related to disease stage was not included, as some subjects were newly diagnosed, whereas others had been diagnosed for several years. Thus, the results could have been affected by using subjects with a range of mild to severe disease. While subjects taking other polysaccharide dietary supplements and certain medications were excluded, we did not omit other agents, such as antioxidants, polyphenols, or other phytochemicals. Thus, those nutrients might have confounded the results. Diet was not evaluated in this study, particularly fats, which could have also confounded the results, even in a 90-day period. As fats, such as palmitic acid, which are common in the human diet, are used to create NAFLD-type models in animals [54], it would be important to know if total energy, macronutrient content, or other micronutrients contributed to the results in this study. Physical exercise also was not assessed in this study, and this variable could also have been a confounding factor, as regular exercise in mice proliferates IL-18 secretion [55]. The study included a relatively small sample size in a 90-day intervention period on a modest dose of RBAC (1 g/day). The decision to use 1 g/day was due in part to the results of our prior study in healthy adults [15], but patients with a complex disease such as NAFLD may require a higher dose to achieve more significant results.

## 5. Conclusions

Conventional medicine has provided limited efficacious treatment for NAFLD patients. Currently, the only effective remedy for reducing fat in the liver is through weight loss with consistent hypocaloric eating. Nonetheless, compared to pharmacological treatments that offer minimal benefits, yet are associated with clear side effects, an efficacious dietary supplement would provide the cells with raw materials to support innate biochemical and physiological optimization and restoration. RBAC has historically conferred immunomodulatory benefits in humans, animals, and cells on a wide variety of outcome variables, for example, NK and dendritic cells, IL-6, TNF-*α*, IFN-*γ*, and VEGF, to name only a few [13, 15, 56, 57]. Given our previous work in healthy adults showing that RBAC enhances NK cell cytotoxicity and improves the overall inflammatory profile according to a number of cytokines and growth factors [15], we chose to evaluate its effect on several biomarkers relevant to NAFLD.

In this study, RBAC caused no adverse effects, and no subject reported any complications with the intervention. Attrition was minimal, and compliance to the intervention was acceptable at both follow-ups. ALP, a hallmark liver enzyme, significantly improved in response to RBAC. The percentage of eosinophils and monocytes increased at the 90-day follow-up, suggestive of an immunomodulatory response, which is consistent with previous findings on RBAC. Platelets decreased in the RBAC group, which may be another important clinical improvement for this population, as possible modulation of an acute phase reactant. Although statistically nonsignificant, neutrophils, NLR, GGT, and 4-hydroxynonenal all improved in the RBAC group, which have important clinical implications for these patients. ALP and GGT both declined in response to RBAC, which is a clinically significant finding for hepatobiliary observation. IFN-*γ* increased from baseline to 90 days, while IL-18 increased from 45 to 90 days, in the RBAC group in what appears to be a complex relationship among these immune markers that has been documented previously. The overall decrease in IL-18 from baseline to 90 days is consistent with a previous animal study on RBAC.

No other statistically or clinically significant effects were noted in the biomarkers in response to RBAC. Nonetheless, combined with our prior findings on RBAC and those of others showing such potent immunomodulatory activity, the results of the present study support the use of this product for patients with NAFLD as a complement or alternative to conventional medical treatments. RBAC is an all-natural product with no documented side effects and has no known negative interactions with pharmaceuticals or other dietary supplements.

The next step in the evaluation of RBAC would be to extend the current study with perhaps a higher dose and a larger sample size for a longer intervention duration and utilize stricter inclusion criteria to enroll patients with liver biopsy to make the sample more homogenous. In addition, assessing NK cell function, given its relationship to monocytes and the Th-1 immune response, might shed further light on the effect of RBAC in this population. Given the encouraging findings in the current study, additional research is warranted to evaluate RBAC as a treatment option for patients with NAFLD, a complex disease with a mysterious etiology.

## Figures and Tables

**Table 1:** Sociodemographic characteristics of the sample.

Variable	Category	RBAC (*n* = 12)	Placebo (*n* = 11)	Statistic
Age	-	M = 58.1, SD = 15.2, *R* = 21, 75	M = 50.6, SD = 11.6, *R* = 25, 61	*t* = 1.3 (21), *p* = 0.20

Gender	Male	9 (41%)	13 (52%)	*χ* ^2^ = 0.6 (1), *p* = 0.45
	Female	13 (59%)	12 (48%)	

Race/ethnicity	White, non-Hispanic	5 (42%)	1 (9%)	*χ* ^2^ = 5.9 (3), *p* = 0.12
	Black, non-Hispanic	1 (8%)	-	
	Hispanic	4 (33%)	9 (82%)	
	Other	2 (17%)	1 (9%)	

Education	Up to some college	4 (33%)	4 (36%)	*χ* ^2^ = 0.2 (2), *p* = 0.93
	College graduate	3 (25%)	2 (18%)	
	Master's degree or higher	5 (42%)	5 (46%)	

Marital status	Never married	3 (25%)	4 (36%)	*χ* ^2^ = 2.4 (2), *p* = 0.31
	Married	7 (58%)	3 (28%)	
	Divorced	2 (17%)	4 (36%)	

*Note*. M = mean, SD = standard deviation, and *R* = range.

**Table 2:** Liver enzymes, kidney function, lipids, and oxidative stress markers at baseline and 45 and 90 days.

Measure	Reference range	Time	RBAC	Placebo
ALP (U/L)^*∗*^	Male (40–129) Female (35–104)	Baseline	86 ± 37.6 (47, 172)	86.1 ± 31.8 (44, 141)
		45 days	81.2 ± 27.5 (45, 130)	91.8 ± 39.8 (43, 168)
		90 days	80 ± 25.4 (50, 140)	96.8 ± 38.1 (46, 159)

ALT (U/L)	Male (0–40) Female (0–35)	Baseline	39.4 ± 21.9 (12, 74)	57.8 ± 37 (13, 114)
		45 days	35.9 ± 22.9 (11, 83)	59 ± 42.7 (16, 140)
		90 days	38.6 ± 30.5 (13, 118)	55.4 ± 49 (13, 165)

AST (U/L)	Male (0–43) Female (0–32)	Baseline	28.8 ± 10.3 (17, 48)	42.6 ± 20.2 (14, 92)
		45 days	29.2 ± 13.4 (15, 56)	43.3 ± 22.1 (20, 83)
		90 days	30.4 ± 17.9 (17, 81)	44.4 ± 30.3 (19, 120)

AST/ALT ratio	Male (<1) Female (<1)	Baseline	0.86 ± 0.31 (0.58, 1.44)	0.9 ± 0.34 (0.48, 1.39)
		45 days	0.95 ± 0.33 (0.58, 1.54)	0.9 ± 0.36 (0.56, 1.65)
		90 days	0.92 ± 0.32 (0.57, 1.67)	1.02 ± 0.42 (0.52, 1.72)

*γ*-Glutamyl transferase (IU/L)	0–51	Baseline	67.5 ± 80.6 (13, 304)	78.6 ± 87.7 (8, 300)
		45 days	51.4 ± 49.3 (15, 182)	83.1 ± 74.1 (14, 248)
		90 days	46.7 ± 38.1 (13, 126)	98.2 ± 94.6 (15, 291)

Albumin (g/dL)	3.5–5.2	Baseline	4.6 ± 0.3 (4.1, 4.9)	4.5 ± 0.4 (3.6, 4.9)
		45 days	4.5 ± 0.2 (4.3, 4.7)	4.5 ± 0.5 (3.3, 5.2)
		90 days	4.5 ± 0.3 (4, 4.9)	4.6 ± 0.5 (3.5, 5)

Non-HDL cholesterol (mg/dL)	<100 for healthy subjects; <80 for subjects with coronary artery disease	Baseline	142.5 ± 26.7 (81, 184)	146.7 ± 68.8 (36, 275)
		45 days	147.9 ± 33.6 (103, 214)	143.3 ± 65.7 (33, 247)
		90 days	142.8 ± 28.1 (96, 206)	144.6 ± 55.7 (37, 220)

4-Hydroxynonenal (*μ*g/mL)	None	Baseline	121.1 ± 163.6 (6.2, 588.1)	144.5 ± 203 (5.7, 636.9)
		45 days	112.7 ± 150.8 (13.3, 534.7)	252.4 ± 367.4 (5.9, 1035.7)
		90 days	75.9 ± 50.8 (19.4, 157.7)	911.3 ± 2,258.3 (7.9, 6,485.6)

*Note*. Values are mean ± standard deviation (minimum, maximum). ALP: alkaline phosphatase; ALT: alanine transaminase; AST: aspartate transaminase; and HDL: high-density lipoprotein. ^*∗*^Percent change difference (*p* < 0.05) from baseline to 90 days.

**Table 3:** White blood cell subsets and platelets at baseline and 45 and 90 days.

Measure	Reference range	Time	RBAC	Placebo
White blood cells (10^3^/*μ*L)^*∗*^	4.3–10.3	Baseline	6.7 ± 2.7 (3.6, 13.8)	5.5 ± 1.1 (2.5, 6.4)
		45 days	6.5 ± 2.5 (3.8, 12)	5.8 ± 1.5 (4.2, 9)
		90 days	6.7 ± 2.8 (3.4, 13.4)	6 ± 1.6 (2.8, 8.4)

Neutrophils (%)	41–73	Baseline	53.1 ± 9.2 (37.7, 65.7)	56.7 ± 6.1 (45, 67.6)
		45 days	50.8 ± 10.2 (25.8, 64.5)	56.4 ± 5.9 (45.8, 65.3)
		90 days	48.5 ± 10.6 (25.5, 66.9)	56.6 ± 6.1 (47.6, 64.8)

Lymphocytes (%)	19.4–44.9	Baseline	36.8 ± 7.5 (26, 51.2)	32.7 ± 5.7 (22.9, 42.6)
		45 days	37.9 ± 9.6 (23.6, 61.8)	32.8 ± 6.5 (20.2, 42)
		90 days	38.6 ± 9.6 (22.2, 60.1)	32.4 ± 4.9 (29.4, 39)

Neutrophil/lymphocyte ratio	None	Baseline	1.5 ± 0.6 (0.7, 2.5)	1.8 ± 0.5 (1.1, 2.5)
		45 days	1.5 ± 0.6 (0.4, 2.7)	1.8 ± 0.6 (1.1, 3.0)
		90 days	1.4 ± 0.7 (0.4, 3.0)	1.8 ± 0.4 (1.2, 2.3)

Monocytes (%)^+^	5.1–10.9	Baseline	7.3 ± 2.1 (3.5, 11.8)	7.3 ± 4 (3.6, 18.5)
		45 days	7.8 ± 1.2 (5.9, 10.1)	8.1 ± 3.3 (5.5, 16.5)
		90 days	9.1 ± 1.3 (6.9, 11.3)	8.1 ± 4.4 (4.5, 19.4)

Eosinophils (%)^∧*∗*^	0.9–6.0	Baseline	2.5 ± 1.3 (1.2, 4.9)	2.9 ± 1.4 (1.1, 5.5)
		45 days	3 ± 1.2 (2.1, 5.5)	2.2 ± 1.1 (0.5, 4.1)
		90 days	3.3 ± 1.5 (1.6, 6)	2.2 ± 1.2 (0.8, 4.4)

Platelets (10^3^/*μ*L)^*∗*^	156–373	Baseline	254.8 ± 90.5 (180, 511)	196.9 ± 61.9 (81, 292)
		45 days	225.6 ± 46.7 (170, 311)	194.3 ± 65.3 (55, 267)
		90 days	229.5 ± 51.8 (176, 351)	210.4 ± 91.6 (65, 398)

*Note*. Values are mean ± standard deviation (minimum, maximum). ^*∗*^Percent change difference (*p* < 0.05) from baseline to 90 days; ^+^percent change difference (*p* < 0.05) from 45 days to 90 days; ^∧^percent change difference (*p* < 0.05) from baseline to 45 days.

**Table 4:** Cytokines and growth factors at baseline and 45 and 90 days.

Measure	Reference range	Time	RBAC	Placebo
IL-6 (pg/mL)	0.6–2.8	Baseline	1.4 ± 1.4 (0.01, 4.8)	0.7 ± 0.9 (0.01, 2.1)
		45 days	1.4 ± 0.9 (0.01, 2.7)	2.8 ± 1.7 (0.01, 5.2)
		90 days	1.7 ± 0.9 (0.09, 3.3)	1.7 ± 1.3 (0.01, 4.2)

IL-18 (pg/mL)^+^	36.1–257.8	Baseline	446.1 ± 876 (64.5, 3,221)	256.4 ± 106.8 (86.6, 430.2)
		45 days	196.3 ± 64.7 (49.6, 284)	371 ± 176.5 (83.8, 597.1)
		90 days	221.3 ± 44.9 (153.5, 300.2)	263.3 ± 116.2 (75.7, 409.5)

IFN-*γ* (pg/mL)	0–3.0	Baseline	1.3 ± 1.5 (0.01, 5.1)	2.3 ± 2.5 (0.01, 7.8)
		45 days	2.5 ± 1.9 (0.01, 7.4)	2.4 ± 0.9 (1.1, 4.2)
		90 days	2.3 ± 1.5 (0.01, 4.9)	1.9 ± 1.8 (0.01, 4.9)

IL-2 (pg/mL)^*∗*^	1.6–8.3	Baseline	8.4 ± 3.3 (4.2, 13.2)	7.8 ± 6.5 (0.01, 21.2)
		45 days	7.5 ± 3.5 (0.01, 11.7)	7.6 ± 3.1 (4.9, 14.1)
		90 days	6.5 ± 4 (3.8, 18.2)	8.2 ± 3.8 (4.5, 16.4)

TNF RII (pg/mL)	714.4–1,145.5	Baseline	136.2 ± 443.2 (369.7, 1,940)	1259.9 ± 709.5 (391.9, 2,749.3)
		45 days	991.1 ± 277.5 (624.1, 1,530.6)	3449.2 ± 7,339.7 (596.1, 22,998.6)
		90 days	1029.8 ± 275.5 (649.1, 1,564.1)	950.3 ± 506.4 (329.9, 1,753.1)

IL-17 (pg/mL)	1.4–5.0	Baseline	7.6 ± 8.2 (3.3, 33.4)	6.3 ± 3.7 (3.6, 16.9)
		45 days	7.4 ± 6.2 (3.5, 25.8)	7.8 ± 4.7 (2.2, 16.7)
		90 days	5.5 ± 3.7 (0.1, 10.9)	4.9 ± 3.3 (1.1, 10.2)

*Note*. Values are mean ± standard deviation (minimum, maximum). IL: interleukin; IFN: interferon; and TNF: tumor necrosis factor. ^*∗*^Percent change difference (*p* < 0.05) from baseline to 90 days; ^+^percent change difference (*p* < 0.05) from 45 days to 90 days.

## Data Availability

Readers of the article can contact the corresponding author to gain access to the data file of the study.

## References

[1] Parnell J. A., Raman M., Rioux K. P., Reimer R. A. (2012). The potential role of prebiotic fibre for treatment and management of non-alcoholic fatty liver disease and associated obesity and insulin resistance.

[2] Byrne C. D., Olufad R., Bruce K. D., Cagampang F. R., Ahmed M. H. (2009). Metabolic disturbances in non-alcoholic fatty liver disease.

[3] Browning J. D., Szczepaniak L. S., Dobbins R. (2004). Prevalence of hepatic steatosis in an urban population in the United States: impact of ethnicity.

[4] Preiss D., Sattar N. (2008). Non-alcoholic fatty liver disease: an overview of prevalence, diagnosis, pathogenesis and treatment considerations.

[5] Neuschwander-Tetri B. A., Caldwell S. H. (2003). Nonalcoholic steatohepatitis: summary of an AASLD Single Topic Conference.

[6] Dowman J. K., Armstrong M. J., Tomlinson J. W., Newsome P. N. (2011). Current therapeutic strategies in non-alcoholic fatty liver disease.

[7] Patel A. A., Torres D. M., Harrison S. A. (2009). Effect of weight loss on nonalcoholic fatty liver disease.

[8] Musso G., Gambino R., Cassader M., Pagano G. (2010). A meta-analysis of randomized trials for the treatment of nonalcoholic fatty liver disease.

[9] Zheng S., Sanada H., Dohi H., Hirai S., Egashira Y. (2012). Suppressive effect of modified arabinoxylan from rice bran (MGN-3) on D-galactosamine-induced IL-18 expression and hepatitis in rats.

[10] Zheng S., Sugita S., Hirai S., Egashira Y. (2012). Protective effect of low molecular fraction of MGN-3, a modified arabinoxylan from rice bran, on acute liver injury by inhibition of NF-*κ*B and JNK/MAPK expression.

[11] Ghoneum M., Matsuura M. (2004). Augmentation of macrophage phagocytosis by modified arabinoxylan rice bran (MGN-3/Biobran).

[12] Tazawa K., Namikawa H., Oida N. (2000). Scavenging activity of MGN-3 (arabinoxylane from rice bran) with natural killer cell activity on free radicals.

[13] Cholujova D., Jakubikova J., Czako B. (2013). MGN-3 arabinoxylan rice bran modulates innate immunity in multiple myeloma patients.

[14] Ghoneum M. (2016). From bench to bedside: The growing use of arabinoxylan rice bran (MGN-3/Biobran) in cancer immunotherapy.

[15] Ali K., Melillo A., Leonard S., Asthana D., Woolger J., Wolfson A. (2012). An open-label, randomized clinical trial to assess the immunomodulatory activity of a novel oligosaccharide compound in healthy adults.

[16] Paquissi F. C. (2016). Immune imbalances in non-alcoholic fatty liver disease: From general biomarkers and neutrophils to interleukin-17 axis activation and new therapeutic targets.

[17] Paquissi F. C. (2016). The role of inflammation in cardiovascular diseases: The predictive value of neutrophil–lymphocyte ratio as a marker in peripheral arterial disease.

[18] Proctor M. J., McMillan D. C., Morrison D. S., Fletcher C. D., Horgan P. G., Clarke S. J. (2012). A derived neutrophil to lymphocyte ratio predicts survival in patients with cancer.

[19] Broderick G., Katz B. Z., Fernandes H. (2012). Cytokine expression profiles of immune imbalance in post-mononucleosis chronic fatigue.

[20] Schwenger K. J. P., Allard J. P. (2014). Clinical approaches to non-alcoholic fatty liver disease.

[21] Eslamparast T., Eghtesad S., Poustchi H., Hekmatdoost A. (2015). Recent advances in dietary supplementation, in treating non-alcoholic fatty liver disease.

[22] Del Ben M., Polimeni L., Baratta F., Pastori D., Angelico F. (2017). The role of nutraceuticals for the treatment of non-alcoholic fatty liver disease.

[23] Valenti L., Riso P., Mazzocchi A., Porrini M., Fargion S., Agostoni C. (2013). Dietary anthocyanins as nutritional therapy for nonalcoholic fatty liver disease.

[24] Gopal D. V., Rosen H. R. (2000). Abnormal findings on liver function tests: Interpreting results to narrow the diagnosis and establish a prognosis.

[25] Khodadoostan M., Shariatifar B., Motamedi N., Abdolahi H. (2016). Comparison of liver enzymes level and sonographic findings value with liver biopsy findings in nonalcoholic fatty liver disease patients.

[26] Ghoneum M., Badr El-Din N. K., Abdel Fattah S. M., Tolentino L. (2013). Arabinoxylan rice bran (MGN-3/Biobran) provides protection against whole-body *γ*-irradiation in mice via restoration of hematopoietic tissues.

[27] Ghoneum M., Gollapudi S. (2006). Su.94. Effect of Modified Arabinoxylan from Rice Bran (Mgn-3/Biobran) On Human Neutrophils and Monocyte Functions In Vitro.

[28] Badr El-Din N. K., Abdel Fattah S. M., Pan D., Tolentino L., Ghoneum M. (2016). Chemopreventive Activity of MGN-3/Biobran Against Chemical Induction of Glandular Stomach Carcinogenesis in Rats and Its Apoptotic Effect in Gastric Cancer Cells.

[29] Brenner C., Galluzzi L., Kepp O., Kroemer G. (2013). Decoding cell death signals in liver inflammation.

[30] Rolo A. P., Teodoro J. S., Palmeira C. M. (2012). Role of oxidative stress in the pathogenesis of nonalcoholic steatohepatitis.

[31] Okazaki I., Noro T., Tsutsui N. (2014). Fibrogenesis and carcinogenesis in nonalcoholic steatohepatitis (NASH): Involvement of matrix metalloproteinases (MMPs) and tissue inhibitors of metalloproteinase (TIMPs).

[32] Hutchison R. E., Schexneider K. I., McPherson R. A., Pincus M. R. (2017). Leukocytic disorders.

[33] Chernecky C. C., Berger B. J., Chernecky C. C., Berger B. J. (2013). Differential leukocyte count (diff) - peripheral blood.

[34] Bahcecioglu I. H., Yalniz M., Ataseven H. (2005). Levels of serum hyaluronic acid, TNF-*α* and IL-8 in patients with nonalcoholic steatohepatitis.

[35] Macek Jilkova Z., Afzal S., Marche H. (2016). Progression of fibrosis in patients with chronic viral hepatitis is associated with IL-17+ neutrophils.

[36] Alkhouri N., Morris-Stiff G., Campbell C. (2012). Neutrophil to lymphocyte ratio: a new marker for predicting steatohepatitis and fibrosis in patients with nonalcoholic fatty liver disease.

[37] Abdel-Razik A., Mousa N., Shabana W. (2016). A novel model using mean platelet volume and neutrophil to lymphocyte ratio as a marker of nonalcoholic steatohepatitis in NAFLD patients: Multicentric study.

[38] Chernecky C. C., Berger B. J., Chernecky C. C., Berger B. J. (2013). Gamma-glutamyltranspeptidase (GGTP, gamma-glutamyltransferase) - blood.

[39] Pratt D. S., Feldman M., Friedman L. S., Brandt L. J. (2016). Liver chemistry and function tests.

[40] Burtis C. A., Ashwood E. R. (1994).

[41] Lum G., Gambino S. R. (1972). Serum gamma-glutamyl transpeptidase activity as an indicator of disease of liver, pancreas, or bone..

[42] Scharschmidt B. F., Goldberg H. I., Schmid R. (1983). Approach to the Patient with Cholestatic Jaundice.

[43] Siddique A., Kowdley K. V. (2012). Approach to a Patient with Elevated Serum Alkaline Phosphatase.

[44] Zhong H., Yin H. (2015). Role of lipid peroxidation derived 4-hydroxynonenal (4-HNE) in cancer: Focusing on mitochondria.

[45] Selley M. L. (1998). (E)-4-Hydroxy-2-nonenal may be involved in the pathogenesis of Parkinson's disease.

[46] Selley M. L., Close D. R., Stern S. E. (2002). The effect of increased concentrations of homocysteine on the concentration of (E)-4-hydroxy-2-nonenal in the plasma and cerebrospinal fluid of patients with Alzheimer's disease.

[47] Serviddio G., Bellanti F., Vendemiale G. (2013). Free radical biology for medicine: learning from nonalcoholic fatty liver disease.

[48] Gasparyan A. Y., Ayvazyan L., Mikhailidis D. P., Kitas G. D. (2011). Mean platelet volume: a link between thrombosis and inflammation?.

[49] Tefferi A., Barbui T. (2017). Polycythemia vera and essential thrombocythemia: 2017 update on diagnosis, risk-stratification, and management.

[50] Slaats J., ten Oever J., van de Veerdonk F. L., Netea M. G., Bliska J. B. (2016). IL-1*β*/IL-6/CRP and IL-18/ferritin: Distinct Inflammatory Programs in Infections.

[51] van de Veerdonk F. L., Netea M. G., Dinarello C. A., Joosten L. A. B. (2011). Inflammasome activation and IL-1*β* and IL-18 processing during infection.

[52] Chaix J., Tessmer M. S., Hoebe K. (2008). Cutting edge: priming of NK cells by IL-18.

[53] Nakanishi K., Yoshimoto T., Tsutsui H., Okamura H. (2001). Interleukin-18 regulates both Th1 and Th2 responses.

[54] Cai C., Zhu X., Li P. (2017). NLRP3 Deletion Inhibits the Non-alcoholic Steatohepatitis Development and Inflammation in Kupffer Cells Induced by Palmitic Acid.

[55] Shirato K., Imaizumi K., Sakurai T., Ogasawara J., Ohno H., Kizaki T. (2017). Regular Voluntary Exercise Potentiates Interleukin-1 *β* and Interleukin-18 Secretion by Increasing Caspase-1 Expression in Murine Macrophages.

[56] Ghoneum M., Agrawal S. (2014). Mgn-3/biobran enhances generation of cytotoxic CD8+ T cells via upregulation of dec-205 expression on dendritic cells.

[57] Ghoneum M. (1998). Enhancement of human natural killer cell activity by modified arabinoxylane from rice bran (BIOBRAN).

